# MRX87 family with *Aristaless X *dup24bp mutation and implication for polyAlanine expansions

**DOI:** 10.1186/1471-2350-8-25

**Published:** 2007-05-04

**Authors:** Carmela Laperuta, Letizia Spizzichino, Pio D'Adamo, Jlenia Monfregola, Antonio Maiorino, Angela D'Eustacchio, Valerio Ventruto, Giovanni Neri, Michele D'Urso, Pietro Chiurazzi, Matilde Valeria Ursini, Maria Giuseppina Miano

**Affiliations:** 1 Institute of Genetics and Biophysics "Adriano Buzzati Traverso" CNR, Naples, Italy; 2 Catholic University of Rome, Rome, Italy; 3 Telethon Institute of Genetics and Medicine, TIGEM, Naples, Italy; 4 C.A.R.S.I.C Institute, Venafro, Italy

## Abstract

**Background:**

Cognitive impairments are heterogeneous conditions, and it is estimated that 10% may be caused by a defect of mental function genes on the X chromosome. One of those genes is *Aristaless related homeobox *(*ARX*) encoding a polyA-rich homeobox transcription factor essential for cerebral patterning and its mutations cause different neurologic disorders. We reported on the clinical and genetic analysis of an Italian family with X-linked mental retardation (XLMR) and intra-familial heterogeneity, and provided insight into its molecular defect.

**Methods:**

We carried out on linkage-candidate gene studies in a new MRX family (MRX87). All coding regions and exon-intron boundaries of ARX gene were analysed by direct sequencing.

**Results:**

MRX87 patients had moderate to profound cognition impairment and a combination of minor congenital anomalies. The disease locus, MRX87, was mapped between DXS7104 and DXS1214, placing it in Xp22-p21 interval, a hot spot region for mental handicap. An in frame duplication of 24 bp (ARXdup24) in the second polyAlanine tract (polyA_II) in ARX was identified.

**Conclusion:**

Our study underlines the role of ARXdup24 as a critical mutational site causing mental retardation linked to Xp22. Phenotypic heterogeneity of MRX87 patients represents a new observation relevant to the functional consequences of polyAlanine expansions enriching the puzzling complexity of ARXdup24-linked diseases.

## Background

X-linked mental retardation (XLMR) is a heterogeneous genetic condition characterized by variable cognitive handicap with IQ below 70. To date more than 50 XLMR genes have been recognized [1-3]. Each of them accounts for a very small proportion of the affected families with the exception of *FMR1*, whose loss of function mutation causes the Fragile X syndrome, and the *Aristaless X *(*ARX*) gene mutated in several syndromic and non syndromic mentally retarded patients [4-9].

The *ARX *gene (OMIM #300382) was identified as the causative gene in several allelic brain diseases with MR such as i) XLAG or X-linked lissencephaly with abnormal genitalia (OMIM #300215) [10]; ii) Proud syndrome or mental retardation with agenesis of the corpus callosum, microcephaly, limb contractures, scoliosis, coarse faces, tapered digits and urogenital abnormalities (OMIM #30004) [10]; iii) myoclonic epilepsy syndrome (OMIM #300432) [11]; iv) West syndrome or X-linked infantile spasm syndrome with hypsarrhythmia and mental retardation (OMIM #308350) [12]; v) Partington dystonic syndrome (OMIM #309510) [13]; vi) non syndromic X-linked mental retardation (OMIM #300382) [14].

*ARX *encodes the Aristaless-related protein, a bi-functional homeobox transcription factor essential for cerebral patterning and for the maintenance of specific neuronal subtypes in the cerebral cortex [15]. It belongs to the *Q*_50 _*Paired-like *(*Prd-like*) class genes, an ancient family of transcription factors with a key role in the early evolution of the animal head and development of the central nervous system [16]. The ARX protein contains a number of conserved domains, including the two DNA binding domains (Homeobox and Aristaless), and four distinct hydrophobic polyalanine tracts (polyA_I, II, III and IV) with a hypothetical role as transcriptional suppressor [17,18].

The *Arx *knockout mouse is characterized by a small brain with aberrant migration and differentiation of GABAergic interneuron progenitors and altered testes, a complex phenotype similar to the human XLAG syndrome [19,20]. Murine expression studies showed that *Arx *is widespread throughout telencephalic structures implicated in the pathophysiology of learning formation [13,14,20].

*ARX *gene represents a hot spot for mutations in families with cognition disorders because its mutations account for 9.5% of X-linked MR families [7]. The most frequent mutation is c.428_451dup24, also known as *ARX*dup24, a 24 bp duplication in exon 2 resulting in elongation of the second polyalanine tract (polyA_12__II), that alone might account for 6.6% of all XLMR and 41% of families with mutations in *ARX *gene [4-9]. The c.428_451dup24 mutation has never been found in association with severe brain malformations (i.e. XLAG or Proud syndromes). However, variable phenotypic expression is often observed within the same family with c.428_451dup24 [21,22] reinforcing the notion that *ARX *is a pleiotropic gene that, in a diverse genetic context and/or under the influence of modifier genes, controls different aspects of human brain morphogenesis and function.

Here we present the molecular and clinical characterization of a new XLMR family (MRX87) linked to the Xp21 region in which we found the segregation of the c.428_451dup24 associated to intra-familial clinical variability. Our study aims to enrich the clinical and genetic description of mental defects due to polyalanine expansions in Aristaless protein.

## Methods

### Ascertainment of family members

**Figure 1:**
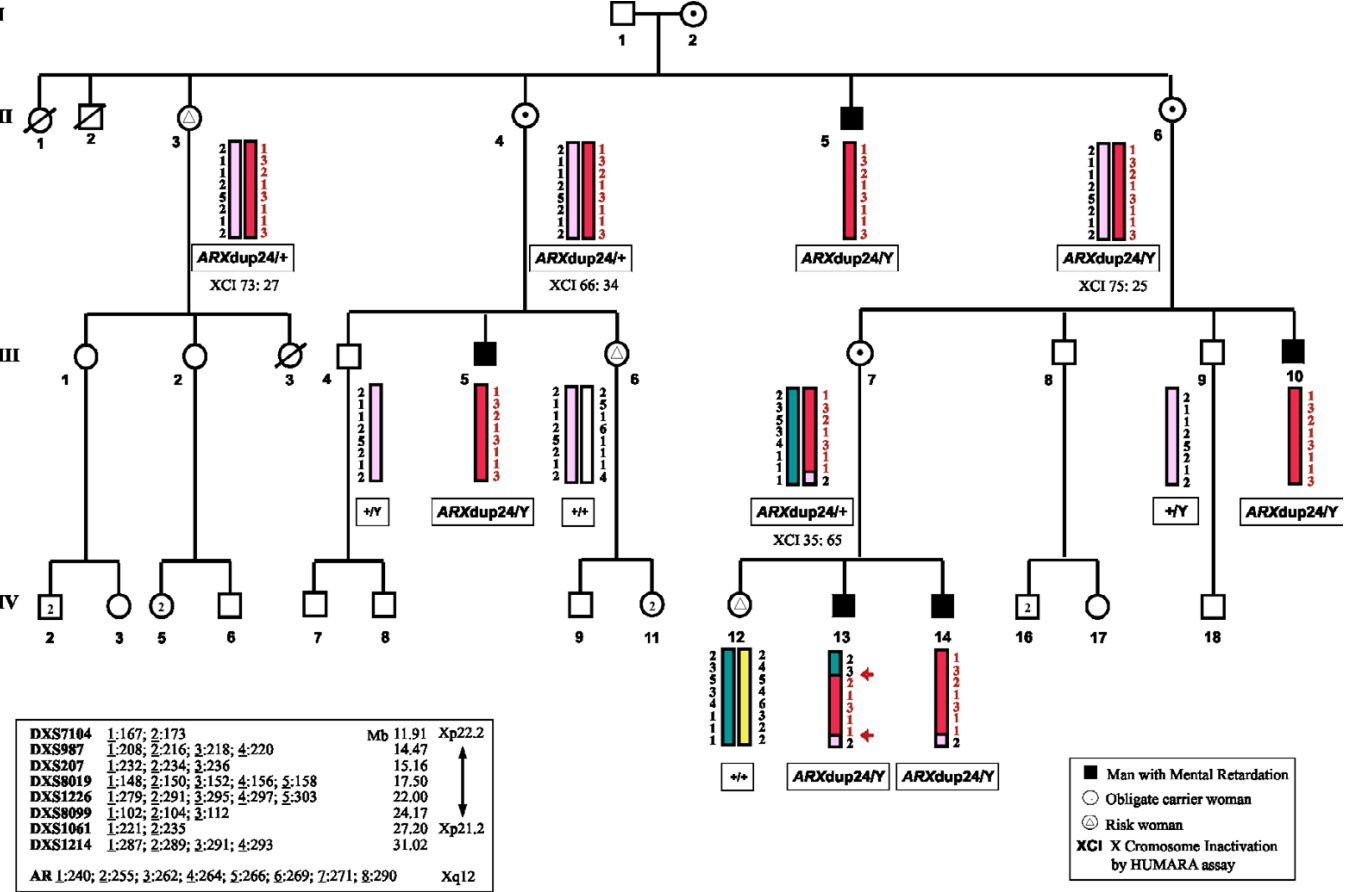
The four-generation family with MRX87 haplotypes for markers in Xp22-p21 and segregation of *ARX *mutation. Thirteen individuals from whom DNA was available were genotyped and linkage analysis was performed. Informative recombinations between the markers DXS987 and DXS207 and between DXS1061 and DXS1214 are indicated with the arrowheads. X disease haplotype (X_d_) is shown in *red*. The X inactivation ratio, obtained by HUMARA (*Human Androgen Receptor *gene, *AR*) assay is indicated for each woman tested. The physical order of the markers analysed is, from telomere (top) to the centromere (bottom), as shown.

Mental retardation was reported in five affected men of a four-generations Italian family (Figure 1). This family includes two affected brothers (IV:13 and IV:14), two affected first cousins (III:5 and III:10) and one affected great uncle (II:5). Peripheral venous blood samples were collected from family members. Informed consent had been obtained. Studies and procedures have been performed with the approval of the ethic committee of the host institutions according to the Helsinki Declaration. Karyotype analysis after G-banding was normal in all family members and molecular analysis of the Fragile X mutation was negative in all patients.

### Linkage analysis

Genomic DNA was isolated from the nucleated peripheral blood leukocytes using the Salting out procedure. A standard set of microsatellite markers on the X chromosome, evenly spaced every 10 cM (ABI PRISM Linkage Mapping Sets vs2, Applied Biosystems) was PCR amplified using conditions already described [3]. Thirteen individuals of the family were genotyped (Figure 1) and PCR products were analysed on automatic sequencer (ABI PRISM 3100, Applied Biosystem). Extra fluorescently labelled primers were synthesized for seven additional polymorphic markers chosen in public databases. Two-point linkage analysis was performed by the MLINK program version 5.1, from the LINKAGE software package [3]. The approved gene symbol MRX87 (Mental Retardation X-Linked 87) was assigned according to the HUGO (Human Genome Organization) nomenclature. X-inactivation status was tested by HUMARA (Human Androgen Receptor Gene) fluorescent assay according to Fimiani *et a*l [23]. XCI patterns were classified as random (XCI ≤ 70%), non random (70% ≤ XCI ≤ 80%) or skewed (XCI ≥ 90%).

### Mutation analysis

All coding exons and the flanking intronic sequences of the *ARX *gene were amplified using DNA from affected and non-affected members of the MRX87 family. Eight primer pairs were used, namely: 1F 5'-CCA ACA CAC ACC CAT CCA T-3' and 1R 5'-CCG AAC ACC AAA CAT CCA A-3' for exon 1; 2aF 5'-CAA GGC GTC GAA GTC TGG TG-3' and 2aR 5'-GTA CGA CTT GCT GCG GCT GA-3', 2bF 5'-CTC CTT CAG GGT GCG GCA GC-3' and 2bR 5'-CCA GCA GCT CCT CCT CGT CG-3', 2cF 5'-CGT CAC GCA CCC GGA GGA GC-3' and 2cR 5'-AGC CCG CTG TCC CTC CCT GG-3' for exon 2; 3F 5'-TGG AGT AGG CCT GCC ATA GA-3' and 3R 5'-CCA ACC CAT CTC TCT CTC TCC-3' for exon 3; 4aF 5'-GCC AAG GGA AGG GAC GGG TA-3' and 4aR 5'-GGT AGG GGC TGA GCG GGT GG-3', 4bF 5'-GAG AAG GCA GGC GCG CAG AC-3' and 4bR 5'-ACT CCT GCC TCC TCC CTG CC-3' for exon 4; 5F 5'-CCT CGG GGA ATA TCT GGA CT-3' and 5R 5'-TTG AGT GGT GCT GAG TGA GG-3' for exon 5. The PCR fragments were sequenced in both directions with the ABI PRISM 3100 DNA sequencer (Applied Biosystem). The 24 base pair duplication in exon 2 was also visualized in patients and carrier women by separating the PCR fragments of amplicon 2a (472 bp) and 2b (409 bp) on a 3% agarose gel.

## Results

### Neurological and physical examination

A diagnosis of MR associated with minor anomalies was made after examination of the patients (II:5, III:5, III:10, IV:13 and IV:14; Figure 1) at the Neurological Center (CARSIC, Venafro, Italy). Careful examination of the patients' phenotypes was performed and the intelligence quotient was assessed by Wechsler Adult intelligence Scale (WAIS). All affected men were born at term after normal pregnancy; no statural growth deficiency was observed. No adverse prenatal events of interest were reported and an extensive metabolic work-up yielded negative results. None of them had convulsion or hand dystonia. All had normal vision and no gonad malformations were recorded.

**Figure 2:**
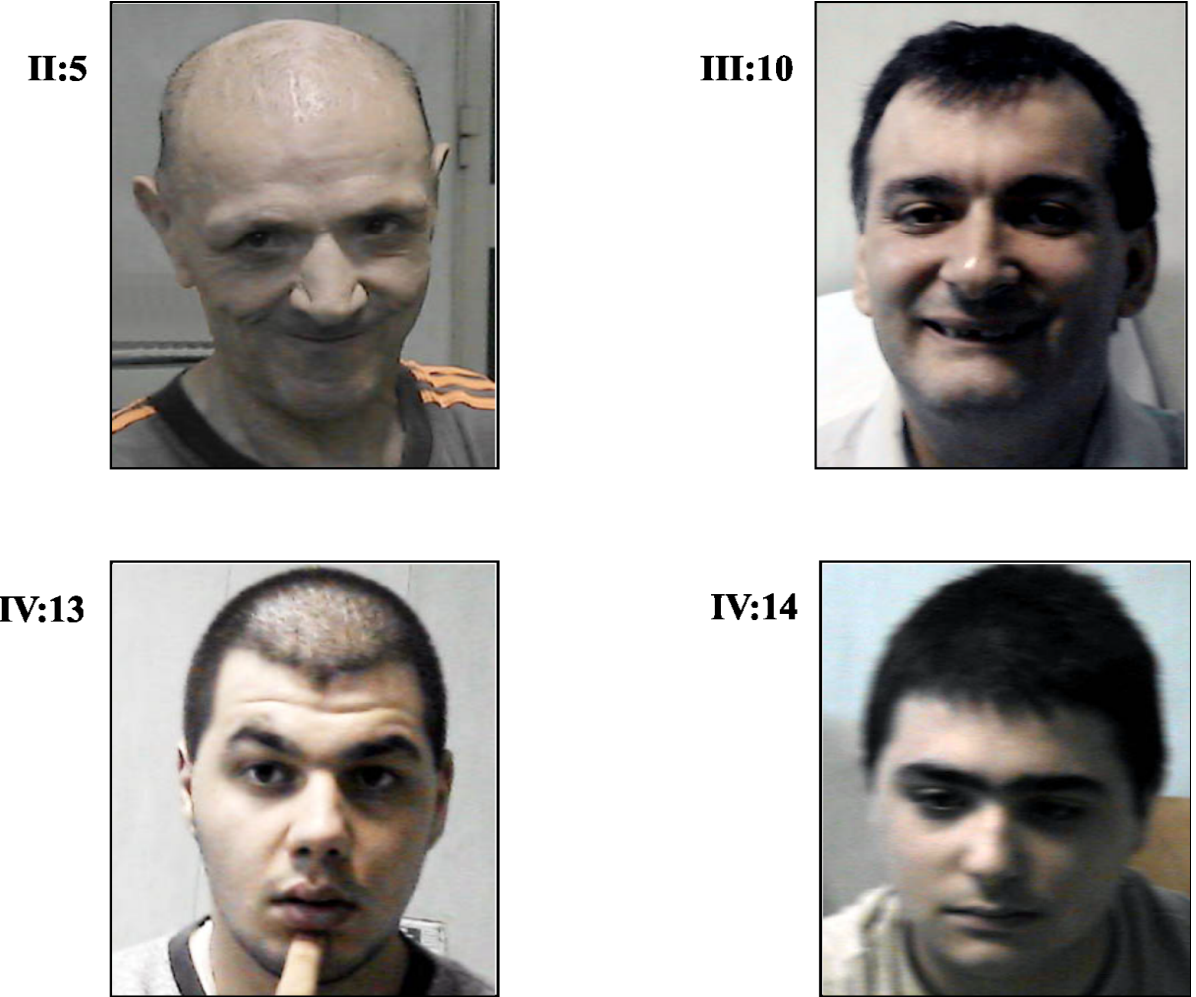
Affected men of MRX87 family with c.428_451dup24 in *ARX *gene. No consistent facial features are present among the patients.

Four of them (II:5, III:10, IV:13 and IV:14) were thoroughly examined and a marked intra-familial heterogeneity was observed. Patients presented a variable cognitive impairment, moderate in IV:13 and IV:14 and severe in II:5, III:5, and III:10. Their clinical evaluation was summarized in Table 1. No dysmorphic signs were observed (Figure 2). In patient II:5, we found pyramidal hypotonia, bilateral Babinski signs and demential behavior. Bilateral neurosensorial deafness and a deficit of the VII cranial nerve were also observed. These signs were not present in the other affected men. However, because II:5 is the oldest affected man in the pedigree, it is possible that these symptoms are age-related and may eventually manifest in the other patients at a later time. Unlike II:5, IV:13 and IV:14, whose MRI examinations did not reveal any structural alterations, patient III:10 showed enlarged subarachnoid spaces and cerebellar tonsils below the foramen magnum (data not shown). No signs of increased intracranial pressure or cortex lesions were detected in this patient.

**Table 1:** Synopsis of MRX87 male patients

***Individual***	***Age***	***Mental handicap***	***Minor anomalies***	***Behavior***	***MRI/CT scan***	***OCF***
**II:5**	67 y	Severe	Pyramidal Hypotonia; Bilateral Babinski sign; Deficit of the VII cranial nerve; Urinary incontinence; Neurosensorial hypoacusis	Demential syndrome	Normal	52 cm
**III:10**	40 y	Severe	Flatfoot	Nd	Cerebellar tonsils below the level of the foramen magnum; Wide subarachnoid spaces	56 cm
**IV:13**	22 y	Moderate	Flatfoot; Urinary incontinence	Nd	Normal	53 cm
**IV:14**	16 y	Moderate	Sialorrhoea; Flatfoot	Language deficit	Normal	54 cm

OCF = Occipital Circumference; Head and neck MRA – Sag T1, axial FLAIR, axial FSE T2. Nd = not determined.

Patient II:5 and patient IV:13 had severe urinary incontinence, a clinical sign often observed in association with MR [24]. Only in patient IV:14, we diagnosed a moderate intellectual handicap associated with a language deficit. Three out of four probands showed a flatfoot deformity (III:10, IV:13 and IV:14), a defect that was not evident in the unaffected men of the family. The obligate carrier women are of normal intelligence and clinically indistinguishable from their non carrier sisters. No carrier mothers recalled serious abnormalities in pregnancy.

### MRX87 was linked to Xp22-Xp21 and is due to a dup24 mutation in the *Aristaless related homeobox X*-chromosome linked gene

Two-point linkage allowed mapping of the MRX87 locus to DXS987 marker in the Xp22-p21 interval. A maximum two point LOD score of 2.43 with no recombination was obtained for three adjacent markers in Xp22-Xp21.1 (DXS207, DXS8019, DXS1226; Figure 1 and Table 2). A double crossover was observed in the patient IV:13 defining the interval between the telomeric (DXS987-DXS207) and the centromeric (DXS1061-DXS1214) markers in which the disease gene was located (Figure 1).

**Table 2:** Two-point LOD scores analysis across the markers DXS7140 and DXS1214 linking MRX87 family to Xp22-p21 interval

***Marker***	0.0*	***0.01***	***0.05***	***0.1***	***0.2***	***0.3***	***0.4***
DXS7104	-2.94	0.02	0.59	0.73	0.69	0.51	0.27
DXS987	1.75	1.72	1.59	1.43	1.09	0.73	0.36
DXS207	2.43	2.38	2.22	2.00	1.54	1.04	0.52
DXS8019	2.35	2.31	2.15	1.94	1.50	1.02	0.52
DXS1226	2.35	2.31	2.15	1.94	1.50	1.02	0.52
DXS8099	1.75	1.72	1.59	1.43	1.09	0.73	0.36
DXS1061	0.35	0.34	0.30	0.26	0.16	0.08	0.02
DXS1214	-7.31	0.31	0.87	0.99	0.90	0.66	0.35

* Recombination fraction.

**Figure 3:**
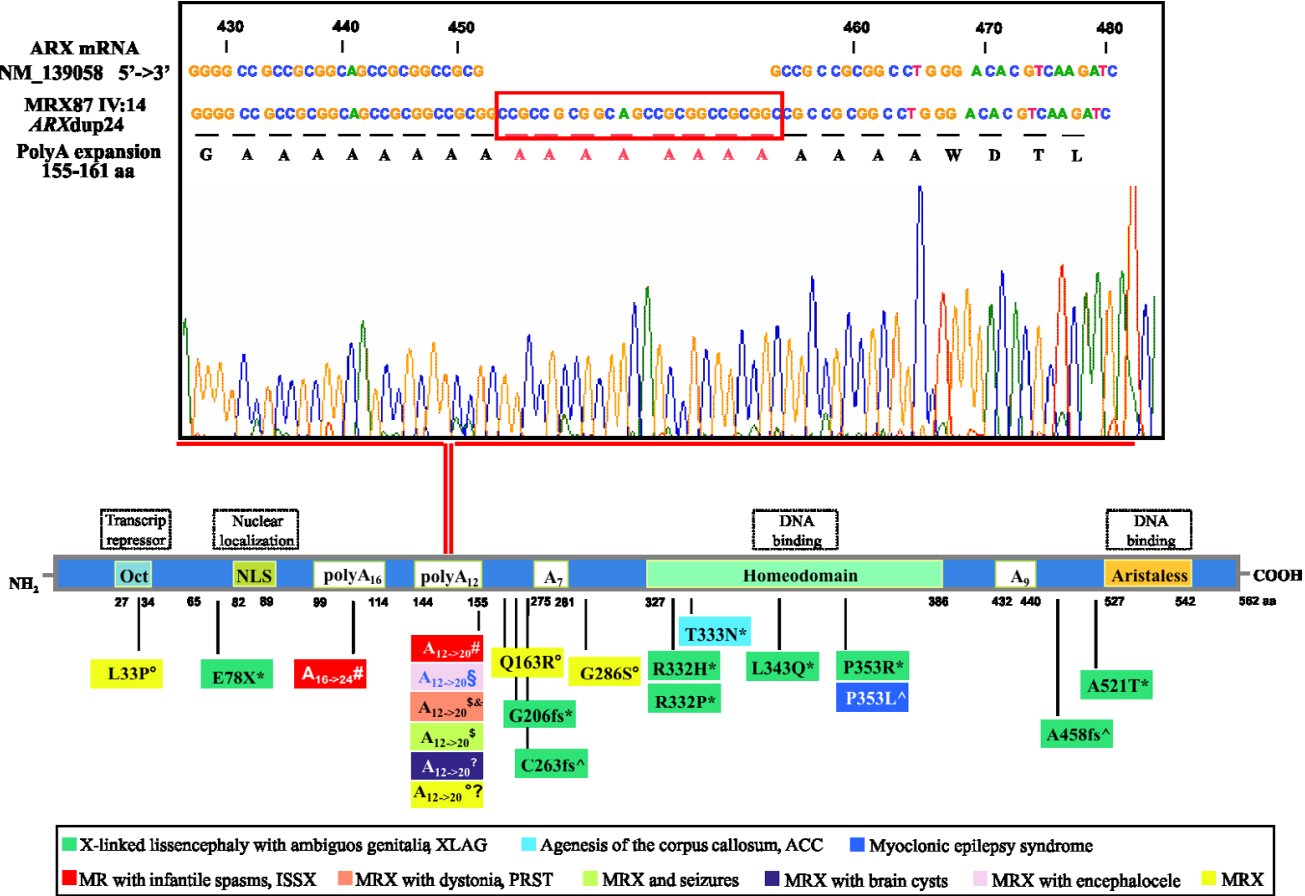
At the top of the figure: Electropherogram of amplicon b of the *ARX *exon 2 in MRX87 IV:14. The box indicates the nucleotide sequence duplicated in the *ARX *gene (c.428_451dup24 also known as *ARX*dup24). At the bottom of the figure: ARX protein functional domains and polyA tracts are shown, next to the various mutations that results in a spectrum of developmental brain phenotypes. *[10], #[13], °[14], ^[19], §[27], ∑[30], ≠[31], $[32], &[33].

This region contains the *ARX *gene, that is, after *FMR1*, the most frequently mutated gene in syndromic and non syndromic X-linked mental retardation [4,5,7]. Therefore, we sequenced *ARX *in the patients of MRX87 family for whom genomic DNA was available. After PCR analysis of the coding exons, the products were directly sequenced. We detected a 24-bp in frame duplication in exon 2, (c.428_451dup24 also known as *ARX*dup24) resulting in the duplication of nucleotides 428–451 (5'-GCCGCCGCGGC AGCCGCGGCCGCG-3'; in GenBank Accession Number NM_139058; Figure 3). The *ARX*dup24 is the most frequent MR mutation found in the *ARX *gene [7]. This duplication is an in frame expansion of the second poly-alanine tract of the ARX protein (amino acids 144–155) from 12 to 20 alanines (Figure 3). The *ARX*dup24 was found in all MRX87 male patients (II:5, III:5, III:10, III:13 and III:14) and was absent in the healthy men (III:4 and III:9). The obligate carriers (II:4, II:6, III:7) and at risk-women (II:3, III:6 and IV:12) were also tested. The *ARX*dup24 was present in all obligate carriers and in II:3, while III:6 and IV:12 did not inherit the mutation (Figure 1). Testing was also performed for individual III:2 because of her mother's status (II:3), ascertaining the absence of c.428_451dup24.

X inactivation analysis (XCI) was performed in the leukocytes of four MRX87 carrier women (II:3, II:4, II:6 and III:7). We analysed the methylation status of the CpG islands of the *AR *gene, using the human *Androgen Receptor *gene fluorescent assay (HUMARA) and excluded the presence of skewed XCI in carrier women (Figure 1). This finding is not completely unexpected because most of the mutations that impair neurocognitive functioning do not confer a selective advantage in leukocytes, as in the case of individuals with Rett syndrome [25], or with Incontinentia Pigmenti [26]. Moreover, X-inactivation was measured in cells of an unaffected tissue (blood) but XCI may be different in the brain or at a critical time during brain development.

## Discussion

This report describes the clinical and molecular findings of an Italian family with the *ARX*dup24 mutation (c.428_451dup24). We linked a new MRX condition, MRX87, to Xp22-Xp21 interval. This is one of the three hot spot regions for X-linked mental retardation containing *ARX*, a gene prominently mutated in both syndromic and non syndromic cognitive impairments. By sequencing its coding region in the affected MRX87 males, we identified the recurrent mutation c.428_451dup24 [7]. This is a duplication of 24 bp in exon 2 that leads to an expansion of the second polyalanine tract (polyA_II) in the ARX protein, from 12 to 20. As far as we know, there are at least 30 published families with c.428_451dup24 showing different clinical presentations including a mild MR to severe MR. Sometimes MR is observed alone, but more often, it is accompanied by a combinations of dystonia, autism, spinocerebellar ataxia, and seizures (Table 3). To this regard, the clinical evaluation of the MRX87 family contributed new elements that enrich the disease spectrum associated to *ARX *mutations. Indeed, patients from our family displayed novel distinctive phenotypical features with a marked clinical intra-variability and variable expression. In particular, a congenital hindbrain hernia, namely Arnold-Chiari like-malformation, was diagnosed in patient III:10. Chiari malformations are composed of a combination of brain stem and cerebellum anomalies. Among the families affected by *ARX*dup24, one was recently described with a boy who, apart from MR, also had a series of congenital anomalies including encephalocele [27], a neurological defect that may occur in association with Arnold-Chiari malformations [28]. Both malformations may be classified as failure of separation of neuro-ectodermal elements from the neural crest [29].

**Table 3:** Summary of clinical data observed in other *ARX*dup24 families

***Families***	***Mental handicap***	***Minor anomalies***	***Behavior***	***MRI/CT scan***	***References***
**MRX54**	Moderate to profound	Long face, thin lips, large ears, epilepsy	Aggressive	Normal	[14]
**P73-MRX**	Moderate	No	Language deficit	Normal	[14]
**P49-MRX**	Moderate	Dystonia	Language deficit, hyperkinesia	Normal	[14]
**MRX36**	Moderate to severe	No	Normal	Normal	[14]
**MRX43**	Moderate to severe	Obesity, large head, epilepsy	Normal	ND	[14]
**MRX76**	Moderate	Wolff-Parkinson- White	Depressive and psychotic features	ND	[14]
**P34-MRX**	Severe	General developmental delays, dystonic hand movements	Language deficit	Normal	[6]
**P104-MRX**	Severe	No	No	Normal	[6]
**P106-MRX**	Moderate	No	Severe language development delay	Normal	[6]
**T37-MRX**	Severe	Long chin and deep-set eyes, strabismus, neonatal hypotonia	Learning and walking difficulties	Normal	[6]
***ARX *family**	Severe	Hypertelorism, broad nasal root, cleft upper lip, growth hormone deficiency	Psychomotor delay	Transsphenoidal encephalocele and agenesis of corpus callosum (ACC) and hypopituitarism	[27]

ND = not determined.

The *ARX*dup24 underlies only a part of the complex phenotypic spectrum of *ARX *mutations. We can distinguish three groups of *ARX *mutations with different outcomes (Figure 3) [10-14,19,27,30-33]: 1. severe mutations causing severe brain patterning malformations due to alterations of the DNA binding domains (HD and Aristaless); 2. expansion in the polyA_I motif causing familial ISSX phenotypes; 3. expansion in the polyA_II motif (c.428_451dup24) causing a spectrum of XLMR conditions with huge inter- and intra-familial heterogeneity. With the exception of the severe *ARX *alterations classified as "loss of function", we cannot establish the functional effect of the polyA expansion mutations. Indeed, *in vitro *data, obtained for only the polyA_I motif, are still controversial [34,35] and no transgenic mice has been produced for each polyA mutation. On the other hand because ARX expansions in both motifs cause varying degree of MR in humans, the functions of the polyA tracts in the ARX protein could be related to the complexity of brain functions such as those controlling memory and learning.

## Conclusion

In conclusion, the identification of a new MRX family linked to Xp22 and carrying the c.428_451dup24 (*ARX*dup24) underlines the high contribution of *ARX *to X linked mental retardation. Furthermore, the clinical findings of the affected members of the MRX87 family enhance the striking phenotypic variability associated with polyA_II expansion.

### Electronic resources

A number of different electronic resources were used in the research for this article. These are listed in the main reference list [36-42].

## Competing interests

The author(s) declare that they have no competing interests.

## Authors' contributions

CL carried out mutational analysis, X inactivation study and participated in the bioinformatic study. LS participated in the nucleotide sequence analysis and mutation identification. PD carried out statistical study. JM participated in the bioinformatic study. AM carried out clinical characterization of the patients. AD performed genotyping. VV identified and diagnosed the patients. GN participated in genetic counselling. MD participated in the design of the study. PC participated in genetic analysis. MVU participated in the design of the study and helped to draft the manuscript. MGM conceptualized, designed the study and drafted the manuscript. All authors read and approved the final manuscript.

## Pre-publication history

The pre-publication history for this paper can be accessed here:


